# Automatic segmentation of ventricular volume by 3D ultrasonography in post haemorrhagic ventricular dilatation among preterm infants

**DOI:** 10.1038/s41598-020-80783-3

**Published:** 2021-01-12

**Authors:** Lionel C. Gontard, Joaquín Pizarro, Borja Sanz-Peña, Simón P. Lubián López, Isabel Benavente-Fernández

**Affiliations:** 1 Department of Condensed Matter Physics, University of Cádiz, 11510 Puerto Real, Spain; 2 Department of Computer Engineering, University of Cádiz, 11519 Puerto Real, Spain; 3 Department of Neurosurgery, Puerta del Mar Hospital, Ana de Viya Avenue 21, 11009 Cádiz, Spain; 4 Department of Pediatrics (Neonatology), Biomedical Research and Innovation Institute of Cádiz (INiBICA) Research Unit, Puerta del Mar Hospital, Ana de Viya Avenue 21, 11009 Cádiz, Spain; 5 Biomedical Research and Innovation Institute of Cádiz (INiBICA) Research Unit, Puerta del Mar University, Ana de Viya 21, 11009 Cádiz, Spain; 6 Foundation for the Development of Neonatal Neurology (Nene), Eloy Gonzalo, nº 5, 28010 Madrid, Spain; 7 Department of Maternal and Child Health and Radiology, School of Medicine, University of Cádiz, Cádiz, Spain

**Keywords:** Diseases of the nervous system, Neurology, Engineering

## Abstract

To train, evaluate, and validate the application of a deep learning framework in three-dimensional ultrasound (3D US) for the automatic segmentation of ventricular volume in preterm infants with post haemorrhagic ventricular dilatation (PHVD). We trained a 2D convolutional neural network (CNN) for automatic segmentation ventricular volume from 3D US of preterm infants with PHVD. The method was validated with the Dice similarity coefficient (DSC) and the intra-class coefficient (ICC) compared to manual segmentation. The mean birth weight of the included patients was 1233.1 g (SD 309.4) and mean gestational age was 28.1 weeks (SD 1.6). A total of 152 serial 3D US from 10 preterm infants with PHVD were analysed. 230 ventricles were manually segmented. Of these, 108 were used for training a 2D CNN and 122 for validating the methodology for automatic segmentation. The global agreement for manual versus automated measures in the validation data (n = 122) was excellent with an ICC of 0.944 (0.874–0.971). The Dice similarity coefficient was 0.8 (± 0.01). 3D US based ventricular volume estimation through an automatic segmentation software developed through deep learning improves the accuracy and reduces the processing time needed for manual segmentation using VOCAL. 3D US should be considered a promising tool to help deepen our current understanding of the complex evolution of PHVD.

## Introduction

Although the survival of premature infants has improved in the past two decades, these infants suffer many potential complications of prematurity, including germinal matrix-intraventricular haemorrhage (GM-IVH). This complication occurs in up to 20–25% of very low birth weight infants (VLBWI) and one third to one half of cases with severe GM-IVH develop post haemorrhagic ventricular dilatation (PHVD). Approximately 20% of the latter will require a permanent ventricle-peritoneal shunt^1^. Moreover, VLBWI with PHVD remain at high risk for neurological problems, including cerebral palsy, epilepsy, cognitive impairment and behavioural issues^2,3^.

Currently, cerebrospinal fluid (CSF) drainage is the cornerstone of treatment for PHVD, but optimal timing for CSF drainage in infants with PHVD is still a matter of debate^4^. Serial cranial ultrasonography measurements of the lateral ventricles play an important role in deciding the optimal timing for intervention and in monitoring ventricular size^5^.

In the current era of technology and artificial intelligence, tools such as 3D ultrasonography (3D US), could improve the evaluation of VLBWI with PHVD.

3D US has the advantages of 2D US with shorter acquisition time and adding the possibility of multi-planar reconstructions, volume rendering and segemtation^6^. It provides an accurate method of ventricular volume estimation, which is based on manually outlined contour definition, a time-consuming process that relies on manufacturer software. This method has fixed settings and does not allow measurement of complex ventricles or those in which the ventricular wall is not clearly outlined (e.g. in the case of parenchymal involvement). If 3D US is to play a role in the diagnosis and monitoring of PHVD in a clinical setting, an accurate and efficient automated segmentation algorithm is crucial.

In this study, we train, evaluate, and validate the application of a deep learning framework (a CNN) for the automatic segmentation of ventricular volume from 3D US of VLBWI with PHVD.

## Results

### Patients and 3D US

We included 10 VLBWI who developed PHVD. The mean birth weight was 1233.1 g (SD 309.4) and mean gestational age was 28.1 weeks (SD 1.6). 4 patients were diagnosed with PHVD following grade III GMH-IVH and six patients had additional parenchymal involvement (IPH or grade IV). A more detailed description of the perinatal variables is shown in Table 1. These ten patients had a median of 18 3D US, with a minimum of six and a maximum of 50.

**Table 1:** Perinatal characteristics of the included patients classified by intervention group.

	Low threshold n = 5	High threshold n = 5	Total n = 10
Gestational age	28.2 (1.64)	27.75 ( 1.5)	28.0 (1.5)
Birth weight	1334 (359.85)	1198.25 (86.13)	1273.67 (259.53)
1-min Apgar	7 [7, 7]	6 [5–6.5]	7 [6, 7]
5-min Apgar	7 [7, 8]	7.5 [6.5–8]	7 [7, 8]
Intraparenchymal haemorrhage	3/5 (60%)	3/5 (60%)	6/10 (55.56%)
Lumbar puncture	5/5 (100%)	2/5 (40%)	7/10 (70%)
Days of life at 1st LP	10 [8–14]	19.5 [15–24]	14 [8–19]
Postmenstrual age at 1st LP	30.1 [28.5–31.5]	29.8 [29.6–30.0]	30 [28.5–31.5]
Reservoir insertion	4/5 (80%)	2/5 (40%)	6/10 (60%)
Days of life at reservoir insertion	12.5 [11–18]	22 [17–27]	15.5 [11–22]
Postmenstrual age at reservoir insertion	29.8 [28.9–31.5]	30.2 [30.2–30.2]	30.2 [29.1–30.4]
Shunt insertion	2/5 (40%)	1/5 (20%)	3/10 (30.0%)
Cognitive	85 (22.73)	90 (10.80)^a^	87.5 (16.69)^a^
Motor	93.25 (20.84)	88.75 (6.65)^a^	91 (14.52)^a^
Language	98 (18.28)	102.5 (10.63)^a^	100.25 (14.05)^a^

Quantitative variables expressed as mean (SD) or median [IQR].

^a^One patient lost to follow-up from the high threshold group.

#### Gold standard annotation: VOCAL

We were able to measure both ventricles in 152 3D US from six patients using VOCAL. In four patients we could only measure one ventricle (the right one in three of them and the left one in the fourth). This was due to limited distinction of the ventricular wall secondary to intraventricular bleed with parenchymal involvement which prevented manual contouring of the ventricle using VOCAL. Right ventricular volume estimation was possible in 132 3D US and the left side was measured in 98 3D US. In total, 230 ventricles were manually segmented using VOCAL. For each manual segmentation, 12 contours were drawn manually, resulting in 2760 contours performed for 230 ventricular volumes. The mean time spent on each 3D US was 20 min, with a total of 76.7 h spent on manual segmentation.

### Reliability

**Figure 1:**
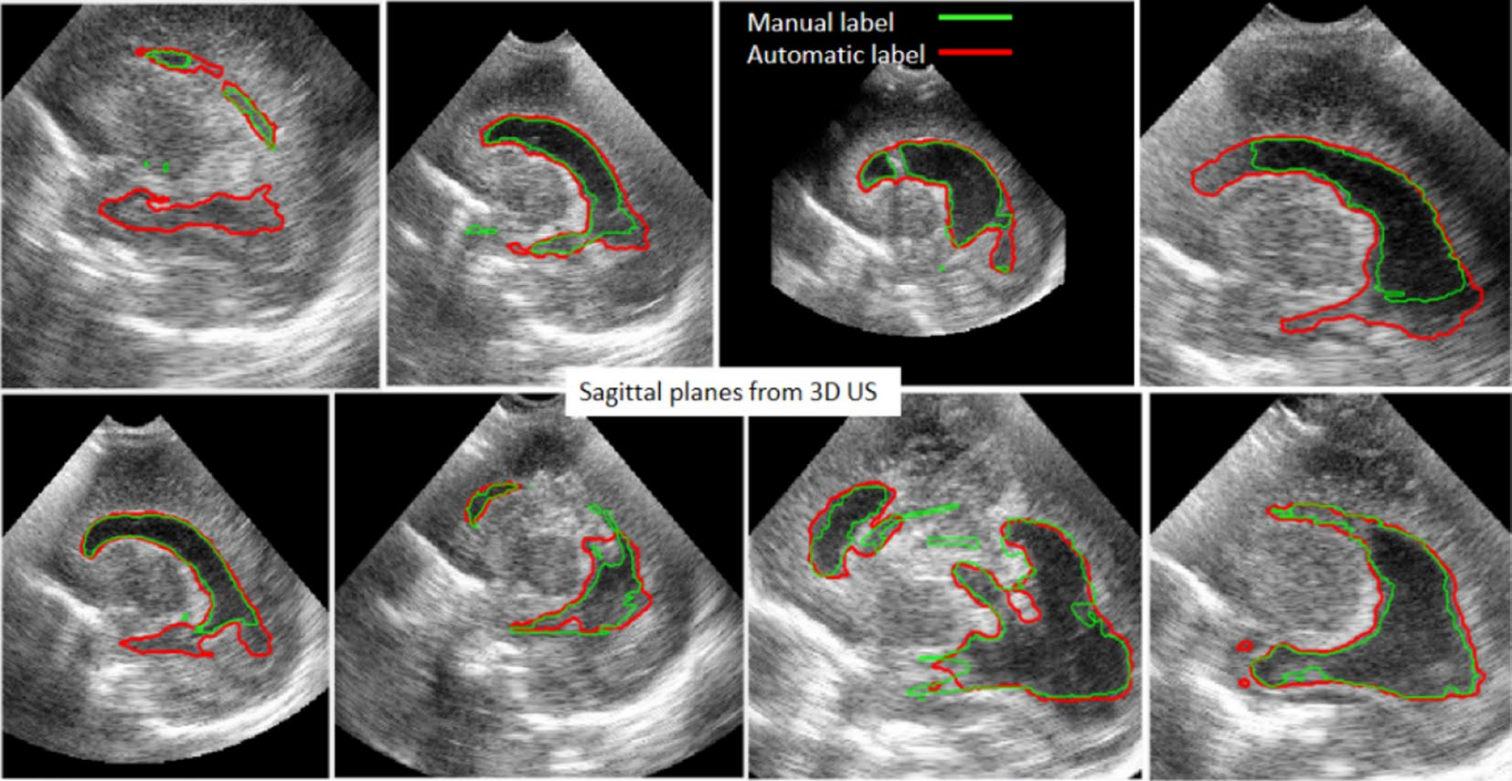
A series of representative sagittal planes extracted from different 3D US of different patients. The contours were measured manually (in green) and using the automatic deep-learning approach (red) one slice at a time. The contours measured automatically were obtained by training the CNN with the manual labels. The large differences seen in several images explain the low value of the IoU metrics. The automatic contours are more accurate.

The reliability of the experiment was measured by the IoU/DSC index of the ventricular class with a mean value of 0.54/0.7. This value indicates a moderate agreement between the learnt 2D labels and the 2D ground truth labels. This is due to the inaccuracies of the manually segmented image labels used for training the CNN, as can be seen in Fig. 1 which shows the overlap between the ground truth and the automatic segmentation. Notably, the figure also demonstrates that the contours obtained automatically often fit better the true contour of the ventricles compared with the manual labels.

**Figure 2:**
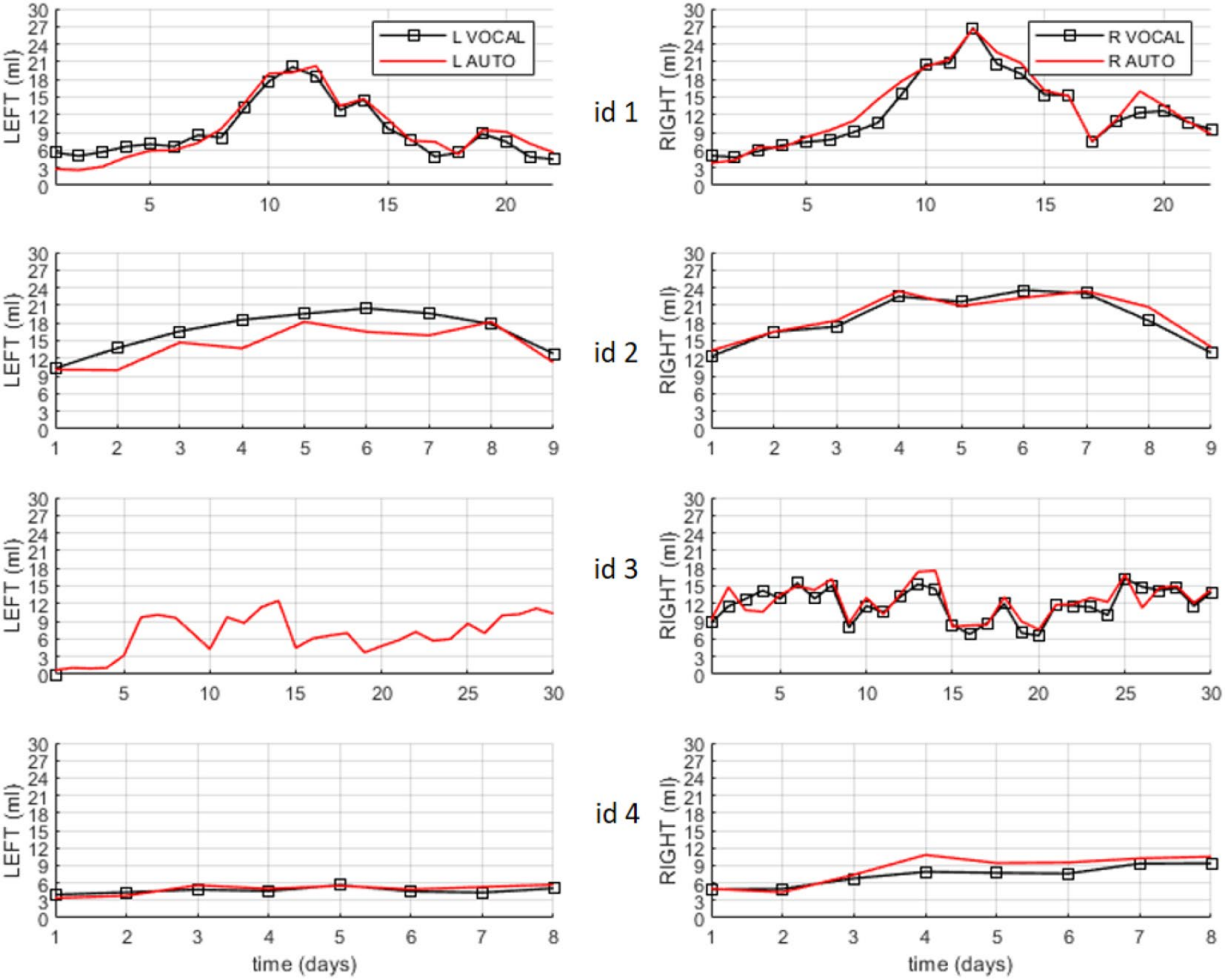
Temporal series acquired over several days of the left (L) and right (R) brain ventricle volumes of four patients (id1–id4). In total, the four series sum 69 3D US. The curves with squared markers are the volumes measured using the gold standard, VOCAL. The curves without markers are the measurements obtained with automatic segmentation. Using VOCAL, there are situations in which the ventricles cannot be measured like the left ventricle of patient id3.

Figure 2 shows the temporal evolution of the volumes of the left and right lateral ventricles of 69 3D US of four patients. In each panel two curves are displayed showing the measurements obtained using either VOCAL or automatic segmentation of the 3D US with the 2D CNN. The average DSC metrics of the volumes of the ventricles was 0.87. The global agreement for VOCAL versus automated measures of the four patients (n = 108 ventricles) was excellent with an ICC of 0.976 (0.965–0.984) (Table 2).

**Table 2:** Reliability of the automated segmentation compared to manual segmentation (“gold standard”) measured by the intraclass correlation coefficient (ICC).

	Training data	Validation data
Global ICC (95% CI)	n = 108 0.976 (0.965–0.984)	n = 122 0.944 (0.874–0.971)
Right ventricle ICC (95% CI)	n = 69 0.963 (0.941–0.977)	n = 63 0.940 (0.882–0.967)
Left ventricle ICC (95% CI)	n = 39 0.987 (0.956–0.994)	n = 59 0.942 (0.80–0.976)
IoU (intersection over union)	0.54	
Dice (mean ± standard deviation [minimum, maximum])	0.8 ± 0.01 [0.63, 0.94]	

### Validation

**Figure 3:**
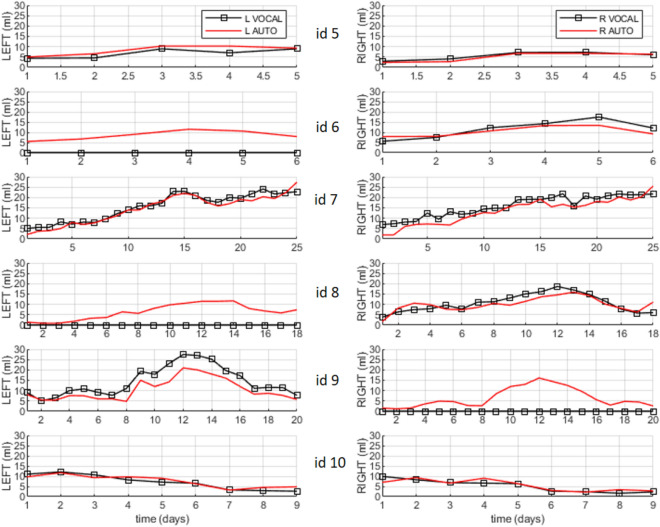
Temporal series acquired over several days of the left (L) and right (R) ventricular volumes of six patients (id5–id10). In total, the six series sum 83 3D US. All of them were used to validate the accuracy of the trained SegNet CNN. The curves with squared markers are the volumes measured using the gold standard, VOCAL. The red curves (without markers) are the measurements obtained with automatic segmentation. Using VOCAL, there are situations in which the ventricles cannot be measured such as the left ventricle of patients id6 and id8, or the right ventricle of id9.

Further validation of the automatic segmentation method is presented in Fig. 3 and Table 2 comparing the ventricular volume measured automatically with the CNN and manually with VOCAL. It shows the trajectories of the volumes of the lateral ventricles on the Y axis and the different 3D US in the X axis of six patients, id5–id10. The average DSC metrics of the ventricles was 0.8.

Automatic ventricular volume estimation was performed in 74 ventricles where manual segmentation had not been possible. The distribution of 3D US segmented by patient is described in Table 1.

#### Passing and Bablok regression

**Figure 4:**
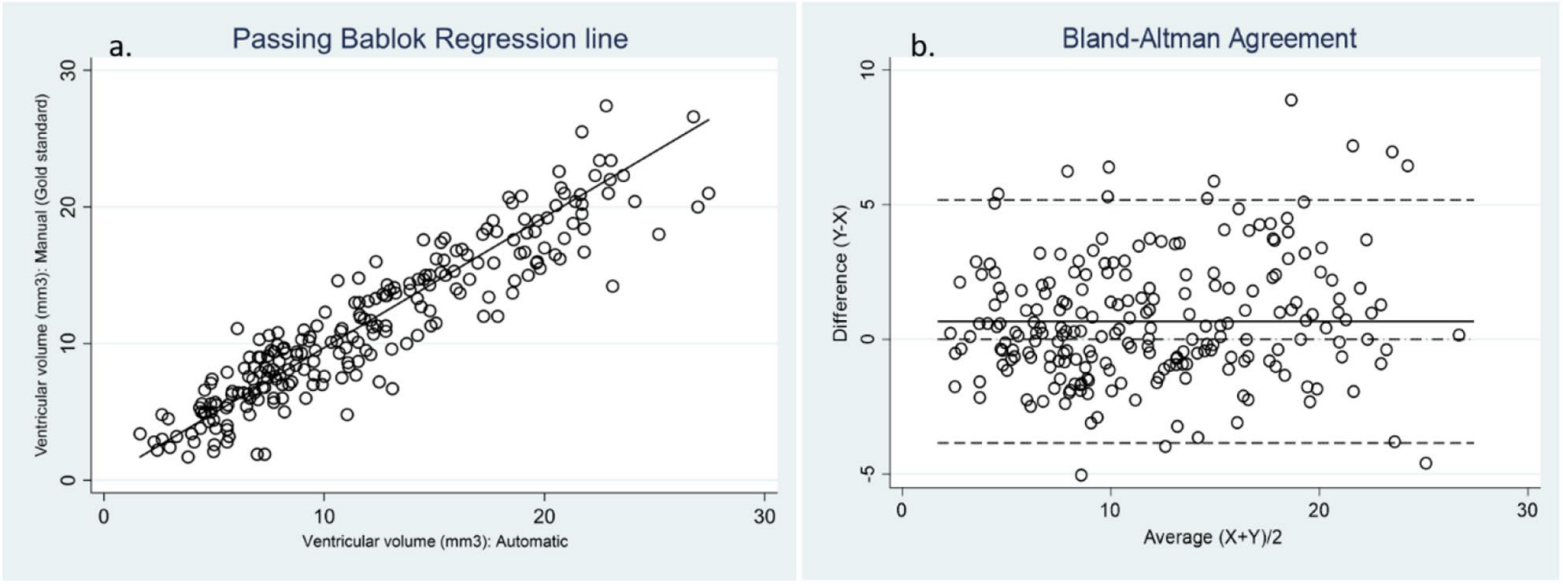
(**a**) The Passing Bablok regression line allows visual inspection of agreement between manual and automatic segmentation. The intercept represents constant differences while the regression line slope represents proportional differences among methods, none of which were significant. (**b**) The observed bias (difference (Y–X)) is 0.61 (SD 2.28) and 94% of the differences are included within the 95% agreement limit (see text for detailed interpretation).

The intercept of Passing–Bablok’s regression Line (Fig. 4a) A = 0.139 (95% CI − 0.311 to 0.756) demonstrates there is no constant difference between the two methods as the 95% CI for intercept includes value zero. The slope B = 0.956 (95% CI 0.905–1.006) for which the 95% CI includes the value of 1, allows us to conclude that there is no proportional difference between the two methods. The Lin’s CCC of Absolute Agreement showed a high correlation (0.9176). We can therefore assume that there is no significant difference between the methods and they can be used interchangeably.

#### Bland Altman method

The observed bias (difference (Y–X)) is close to 0 (0.61 (SD 2.28)) and 94% of the differences are included within the 95% agreement limit with the following limits of agreement (LoA): lower LoA − 3.86 (95% CI − 4.37 to 3.34); upper LoA 5.08 (95% CI 4.57–5.59) with 11 cases (4.78%) being over the limit and 3 cases (1.30%) under the limit (Fig. 4b).

### Clinical application

**Figure 5:**
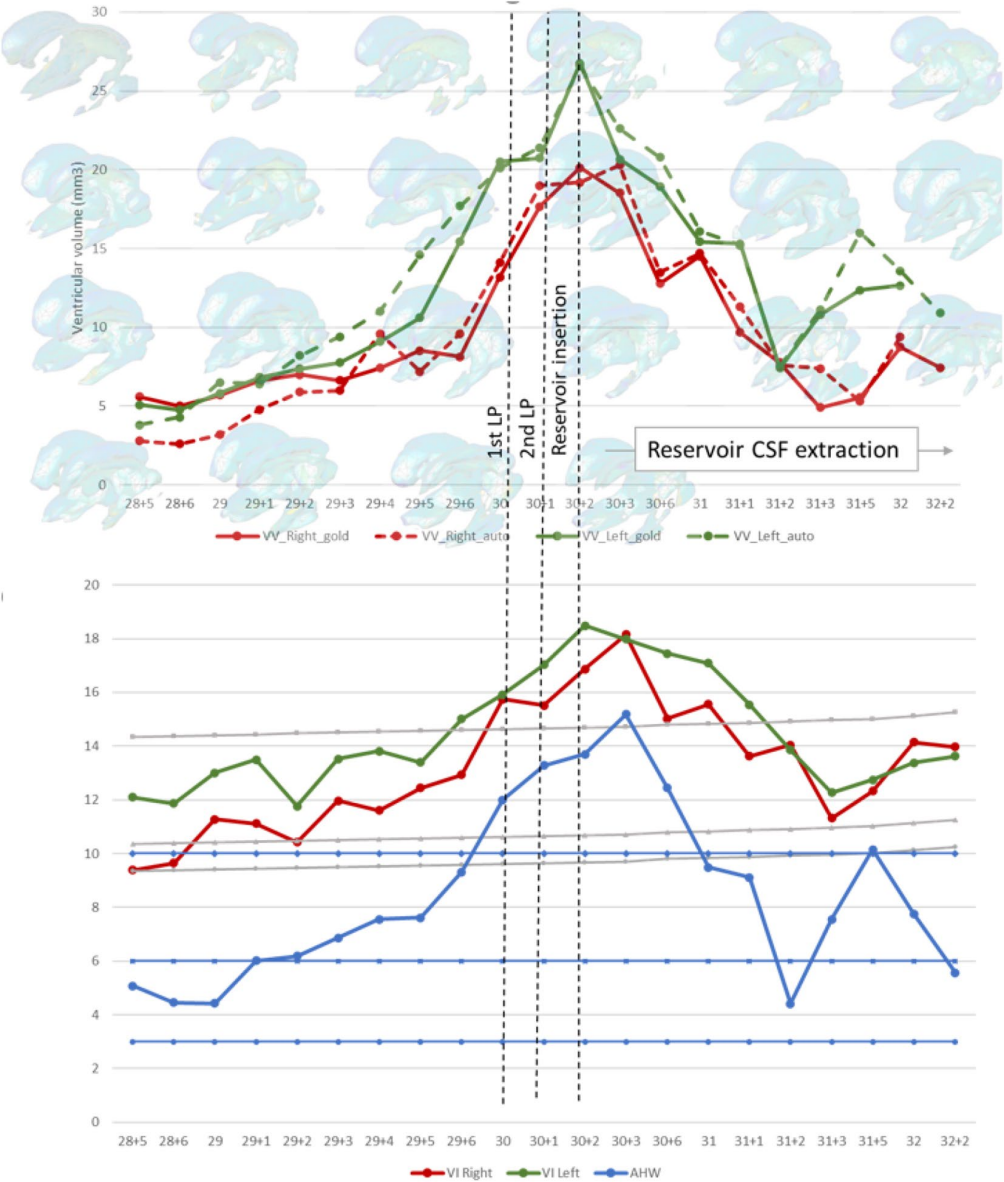
Temporal evolution of ventricular index (VI), anterior horn width (AHW) and ventricular volume in one of the included patients. This patient was randomized to the high threshold intervention group for the ELVIS trial. The volume of the ventricles grows over time. On day 12, an intraventricular catheter was placed to allow CSF removal, allowing for a reduction of ventricular size.

**Figure 6:**
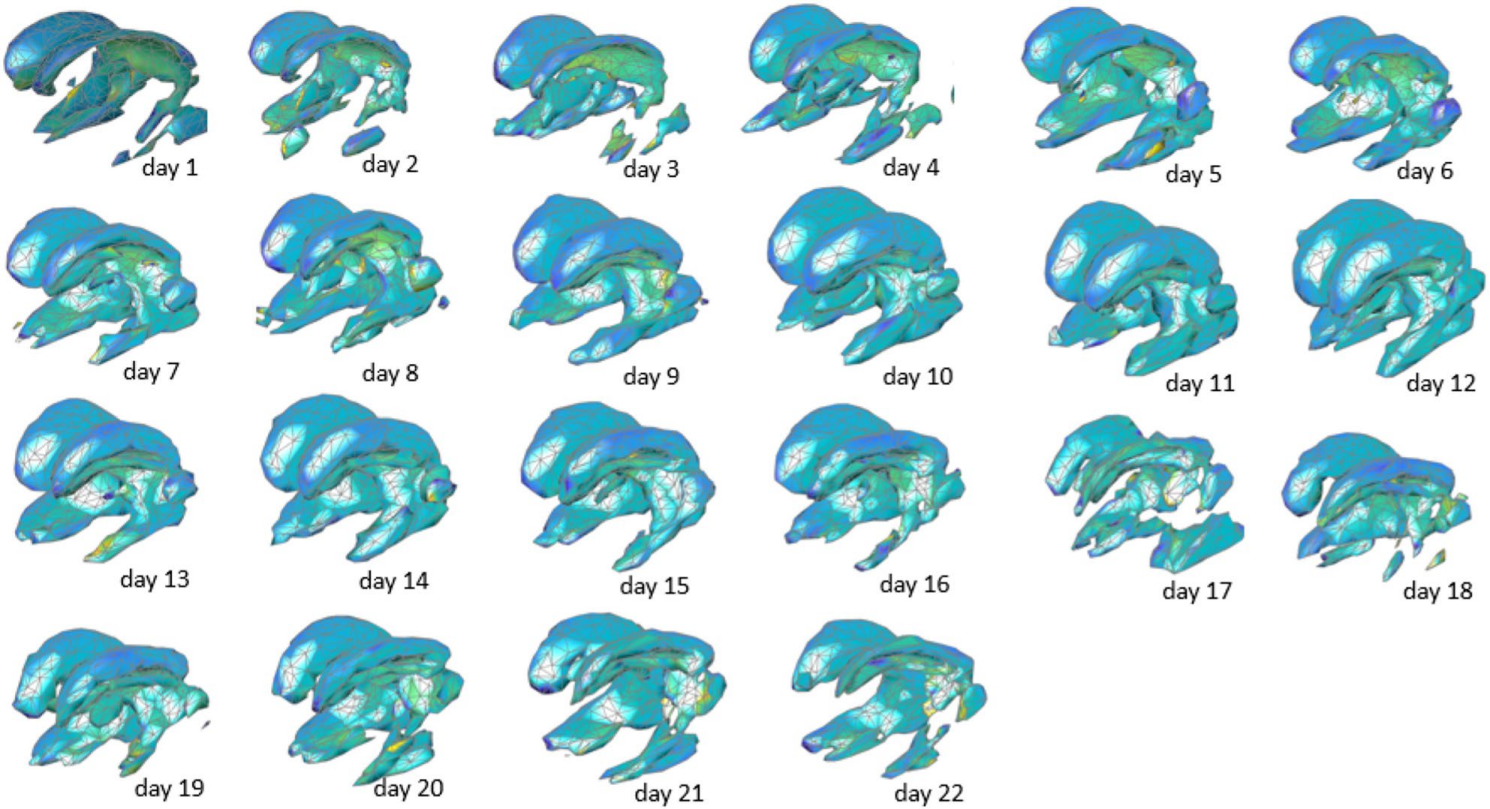
Temporal evolution of ventricular of the ventricular system of one patient (id1) over a period of 22 days measured automatically using deep learning-based segmentation.

In 2D US assessment of the preterm infant, one of the most widely accepted criteria for the diagnosis of PHVD is the Levene ventricular index (VI) reaching the 97th percentile and an anterior horn width (AHW) over 6 mm. Once this threshold was reached, we performed daily 2D US as routine clinical practice in order to detect progressive PHVD. The case shown in Figs. 5 and 6 (patient id 1) was randomized to the high threshold intervention group in the ELVIS trial which considered starting interventions once the VI reached 4 mm over p97 and the AHW was greater than 10 mm. When this threshold was reached, at 30 weeks postmenstrual age, we performed two lumbar punctures on consecutive days. On the third day, a subcutaneous reservoir was placed by the neurosurgeons and thereafter, CSF extraction was performed daily with the amount of CSF and the frequency of punctures determined by daily 2D US. Figure 5 shows how ventricular volume parallels the linear measurements and increases from 5 mm^3^ at the time of diagnosis of PHVD to 26.76 mm^3^ for the left ventricle and 20.12 mm^3^ for the right ventricle when the VI crossed the threshold of p97 + 4 mm (18.48 mm left VI and 16.87 mm right VI) and the AHW was over 10 mm (14.38 mm right, 13.02 mm left) (shown in Fig. 5 as the average AHW of both sides 13.7 mm).

**Figure 7:**
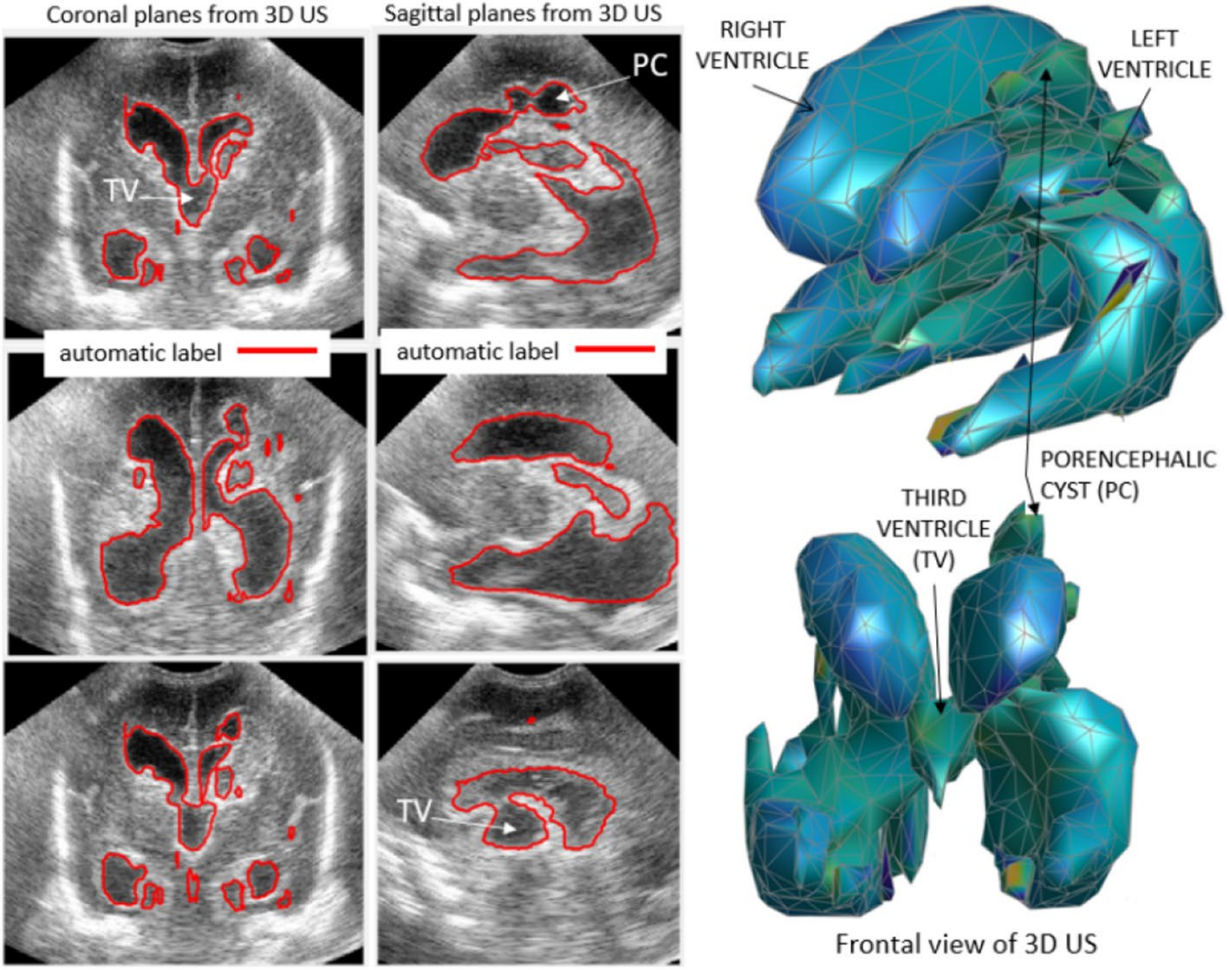
Representative coronal and sagittal planes extracted from a 3D US from a patient with PHVD after grade III GMH-IVH and right intraparenchymal haemorrhage. The contours of the ventricular system measured automatically are drawn in red.

This automated approach enabled us to monitor the evolution of PHVD on a daily basis via 3D US, as well as measure complex ventricles in which neither linear measurements nor manual segmentation could be performed due to intraparenchymal involvement as shown in Fig. 7. We can see the automatic segmentation of the ventricular system from a patient with PHVD after grade III GMH-IVH with right intraparenchymal haemorrhage (id 9). As a porencephalic cyst develops, CSF could also be measured in the periventricular area.

## Discussion

In this study, we report the development of an automatic ventricular volume segmentation tool for 3D US with excellent concordance and reproducibility compared to the gold standard of manual segmentation. We aimed to establish a method that requires minimal to no parameter tuning and still handles routine clinical images including from a wide ranges of ages or with equipment-dependent artefacts or pathologically altered brain tissue.

Although manual segmentation is considered the gold standard, it has some important limitations that limit its widespread use. Image labels of 3D US created by a “perfect labeller” do not exist. At present, the gold standard is created by an expert clinician using VOCAL. Nevertheless, as machine learning methods are improved, the label output by semantic segmentation may be used as labels for further training and ultimately become a standard that does not depend on the subjective input of a human.

Among the most important limitations of manual segmentation are the time-consuming process and the need for software that can be used by clinicians (graphical user interface, GUI). The current commercially available software and user interfaces are powerful in some respects but rigid in others. For example, the software used for manual segmentation (VOCAL, 4d view GE), does not allow segmentation in the absence of ventricular dilatation or in cases of complex ventricular dilatation, since it requires delineation of a single cavity crossed by a vertical axis. In addition, in cases of ventricular wall irregularity where the contour is not well defined and the 3D US image quality is poor, reliable segmentation is not feasible. This is the case shown in Fig. 7 and for several of the volumes shown in Fig. 6, in which VOCAL was not valid for contouring the ventricles.

We have been able to overcome these difficulties with the automatic software developed through the training of a 2D CNN for segmenting 3D US, which both achieved good agreement with VOCAL, and enabled the measurement of ventricular volume in 74 ventricles where VOCAL could not be used. Given the results and the statistical analysis, the automatic software can be considered valid, surpassing what is currently considered the gold standard. An additional limitation of VOCAL is that it performs measurements taking steps of varying degrees of angulation (we chose 15° of rotation to ensure accuracy) and 180° of range, which means that after performing 12 manual delineations of the ventricular contour and thus reaching 180°, the software assumes the symmetry of the structure to be measured, in this case, the lateral ventricle. However, the medial region is not symmetrical to the external region in the ventricular system creating a systematic bias in the commercially available software which should be acknowledged. We have been able to overcome this limitation with the automatic software as it performs a complete evaluation of the ventricular system without the need for tuning any parameters, and the obtained morphometry is closer to the complex anatomy of the ventricular system. Figure 7 shows an example of the complex morphology that the ventricular system of a patient with PHVD can develop. The lateral ventricles may become partitioned and adopt tortuous structures, the third ventricle may become dilated and, in case of parenchymal involvement, as a porencephalic cyst develops, CSF can also be seen in the periventricular area. To the best of our knowledge, the automatic 3D segmentation of complex ventricle surfaces including clots or cysts has not been reported in other studies. Other groups have studied ventricular volume estimation through 3D US in preterm infants without PHVD^7–10^ (Table 3). Csutak et al. measured ventricular volume in healthy neonates from 27 to 41 weeks of gestation proving 3D US automatic scanning by a 3D transducer is superior to free-hand scanning systems, where several 2D US scans are documented by a navigator-controlled transducer^7^. Other authors have previously developed a ventricular volume automatic segmentation software based on US^8,9,11^. Qiu et al.^9^ used a motorized device that mechanically tilts a 2D US transducer and a collection of conventional 2D US is used to build a 3D US. The transducer used in our study is a specific 3D US transducer developed for cranial US and available in our NICU that allows 90° of scan angle while the motorized device used by Qiu et al. reached 60°–72°. A great advantage of deep learning approaches such as the one used in our study is that they are computationally efficient. In terms of computational load, Qiu et al. report a mean processing time of 25 min per 3D US, while our software requires less than one minute, with a less powerful computer. Their algorithm was based on multi atlas registration that requires resource-intensive algorithms such as image registration and image search, thereby increasing the computational time as the size of the atlas increases. In deep-learning approaches like the one used in this study, the computational time at inference time is low and is chiefly dependent on the size of the 3D US.

**Table 3:** Comparison of ventricular volume estimation bey ultrasonography.

	Año	Studied population	Method	Automatic segmentation	Accuracy, reliability and/or gold standard comparation
Csutak et al.	2003	250 healthy infants. Age range from 26 weeks of GA to 6 months	Traced the area of seven to ten sections for each ventricle and multiply by the distance between them	No	No
Kishimoto et al.	2016	26 preterm infants. 11 with moderate to severe IVH	Motorized device. 2D US images used to build a 3D US. Images were manually segmented in parallel sagittal slices 1 mm apart	No	Yes. Compared 3D US to 3D MRI manual segmentation
Qiu et al.	2017	14 preterm infants. 4 with moderate to severe IVH. 70 3D US	Motorized device (same as in Kishimoto et al.). 3D US built from 2D US. Developed a multiatlas segmentation method	Yes	Yes. Mean DSC of 86.4% ± 8.3%
Gontard et al.	2020	10 preterm infants with PHVD. 152 3D US scans	Specific 3d transducer (SVNA5-8B, 5–8 MHz). Developed a deep learning automatic segmentation method	Yes	Yes. Mean DSC 87% in training data. Mean DSC 80% in validation data

*GA* gestational age, *MRI* magnetic resonance imaging, *DSC* dice similarity coefficient, *PHVD* post haemorrhagic ventricular dilatation.

We have shown that the automatic method presented here is reliable by including more 3D US images per patient than other previous studies (230 versus 70 by Qiu et al.). 4 patients of the 14 included in the study by Qiu et al. had moderate to severe GMH-IVH with only three patients requiring any kind of intervention. Our study population is more homogeneous as we included patients with PHVD after moderate or severe GMH-IVH that were included in a randomized trial and were thus more representative of the PHVD population. Finally, the method presented here is robust as it can accurately process 3D US with different voxel sizes, volume dimensions, acquisition conditions (as they were acquired with a handheld scanner), and complex morphologies.

The results demonstrate that after training with thousands of segmented thickened sagittal planes, the CNN has learned to generalize with high accuracy for detecting the contours of the ventricles. Future improvement can involve training with more images and combining the information obtained by training the CNN with axial and coronal thickened slices.

This study has some limitations. The proposed segmentation approach was trained and validated on a limited number of 3D US images. In the future, we plan to train and test our developed segmentation algorithm on a larger number of patient images as this will also help to determine the feasibility of the proposed approach in a clinical context. The small sample size does not allow us to establish a certain ventricular volume as a threshold for diagnosis or intervention in patients with PHVD nor to establish an association between ventricular volume and 2-year outcome. However, this was not the objective of this study, but rather the development of a deep learning method for automatic segmentation that could eventually serve a clinically useful purpose. Another limitation of our study is the use of 2D approach to segment 3D images. To address this limitation, the training set is formed by combining three consecutive 2D images to achieve volumetric information. Future research directions should explore the potential role of ventricular volume monitoring in the diagnostic and therapeutic approach to PHVD.

## Conclusions

3D US based ventricular volume estimation through an automatic segmentation software developed through deep learning achieves results with high accuracy and low processing time. The segmentation method used in this work is a pixel-by-pixel segmentation method, so it is not limited to specific geometric limitations. It is limited only by the quality of the training procedure. The potential clinical use is enhanced with respect to the available software since it also allows measuring complex PHVD. 3D US should be considered an interesting tool to help deepen our current understanding of the complex evolution of PHVD and might prove useful in the future to improve the management and outcomes of very preterm infants with PHVD. Exploring if 3D US and volumetric measurements might have an impact in clinical settings warrants future research.

## Methods

### Patients

We retrospectively reviewed 3D US of VLBWI admitted to the Neonatal Intensive Care Unit (NICU) at Puerta del Mar Hospital who met all of the following inclusion criteria: birth weight less than or equal to 1500 g, gestational age less than or equal to 34 weeks and the development of progressive PHVD. Patients were included if they had more than 5 3D US. These patients participated in the ELVIS trial, a randomized controlled trial (ISRCTN43171322) aimed to compare low vs high-threshold treatment in preterm infants of ≤ 34 weeks’ gestational age with progressive PHVD^12^. Institutional review board approval (Hospital Puerta del Mar research ethics committee) and written parental informed consent were obtained.

We reviewed the 152 3D US scans that were serially performed at different time points with a minimum of 1 day apart to diagnose and monitor PHVD. Transfontanelar 3D US images of the brain were acquired through the 4D option in the 3D/4D Voluson i portable ultrasound system (GE Healthcare, Milwaukee, WI, USA). First, the transducer (SVNA5-8B, 5–8 MHz), using a centre frequency of 6.5 MHz, was situated in the third coronal plane, and the scan angle was settled to 90°. With the transducer fixed in that position, the beam would move from anterior to posterior planes with a maximum total scan time of 40–50 s. The volumes were saved, and the analysis was performed offline with 4D-view version 17.0 software (2001–2019 GE Healthcare Austria GmbH & Co OG). Volumes were also exported from 4D-view format (.vol) to raw data, namely *.nrrd format, widely used in medical imaging and easier to import into other programming packages. The dimensions of the 3D US were different for each patient, ranging from 143 × 183 × 251 to 256 × 237 × 397 voxels with a voxel size of between 0.568 × 0.568 × 0.568 and 0.348 × 0.348 × 0.348 mm^3^, respectively.

### Segmentation of ventricles using deep learning

For the automatic segmentation of the ventricles we used supervised training with deep learning. We carried out the fine-tuning of a 2D convolutional neural network (2D CNN) designed for semantic segmentation called SegNet that has an encoder-decoder architecture based on the VGG16 network^13^. The training of 3D CNNs for 3D US segmentation would require using the full 3D US volumes as input data and a large amount of computing power, which would only be possible if a center has access to datasets with hundreds of 3D US and High Performance Computing platforms. Alternatively, it is possible to use a 2D CNN for consecutively segmenting the slices of a volume at the expense of losing 3D information during training. In this work, we have followed an intermediate route for capturing some 3D information during the training of a 2D CNN consisting of using thickened slices. An advantage with respect to 3D CNNs is that each slice of every 3D US available for training (typically with more than 100 slices each) can be used as input for training the 2D CNN, so that a low number of 3D US can have thousands of images for training the network.Ground truth labelling step.

In order to perform supervised training of the CNN, it is necessary to generate two sets of images: one containing 2D US images and another one with image labels (ground truth images) that are then used in comparing the outputs during training. At present a “perfect labeller” does not exist and the ground truth labels depend on the quality of manual labels generated by an expert radiologist. Manual labelling of the large datasets required for training a CNN is a time-consuming process. We developed a dedicated software tool for accelerating the labelling step of the ventricles by doing a search of an optimum intensity threshold. The algorithm was previously described by our group^14^ and consists of three steps: (1) the 3D US image is preprocessed for noise reduction using a median 3D filter; (2) the filtered dataset is segmented for darker intensities (low density in the brain associated with the fluid in the ventricles) using a global threshold calculated iteratively over the histogram of the 3D volume. In each iteration segmented blobs are classified according to geometrical parameters. Although the method works well in some 3D US, we observed that the volumes calculated were frequently underestimated. Nonetheless, we found the algorithm useful for obtaining a starting segmentation proposal of the ventricles. As a result, for each 3D US we generated a new volume of the same size and in binary format in which all the non-ventricle voxels were set to a value of zero and the voxels of the ventricles to one. Later, each volume was refined by an expert radiologist who corrected the contours manually on each of the 2D images. As a result, we built a training dataset consisting of a total of 4899 sagittal slices and the corresponding ground truth slices from 51 selected 3D US from patients id1–id4.

#### Image preprocessing step

The sagittal slices of the 3D US and the corresponding manually segmented labels require further processing before they can be used for training a CNN as these only accept input images with fixed dimensions, i.e., with the same width (W), height (H), and thickness. The SegNet architecture used in this work accepts as input RGB (colour) images with depth 3 and we chose input dimensions W and H of 200 pixels by 200 pixels for reducing training time and memory usage and by considering the average size of the sagittal images. As the dimensions of the 3D US files used in this work are not uniform, the first mandatory preprocessing step is to correct dimensionality of all the slices used for training. We generated a new training set of RGB images (with depth 3) by combining consecutive US sagittal slices. More specifically, for each monochrome sagittal slice *s* with height H, width W and thickness 1 we generated a new coloured (RGB) sagittal slice *s’* of size H × W and thickness 3, by setting the slice *s *− 1 to the channel R, the slice *s* to the channel G and the slice *s* + 1 to the channel B. Using this approach, the training of the CNN benefits from both the light computation requirement of 2D methods and the ability to capture contextual information of 3D methods^15^.

The input of the CNN used here contains a depth of 3 but its output (the labelled pixels) has a depth of 1. Therefore, we generated new ground truth labels by compressing every three consecutive image labels into one single-channel image by applying a binary-type OR operation at the pixel level. Next, we resized the H and W of all the RGB images and ground truth labels by padding the images with zeros to match the size of the input of the CNN (200 × 200 pixels). Finally, we applied denoising to all of the images using a median filtering with a kernel size of [1, 3, 3].

#### Balance classes using class weighting

**Figure 8:**
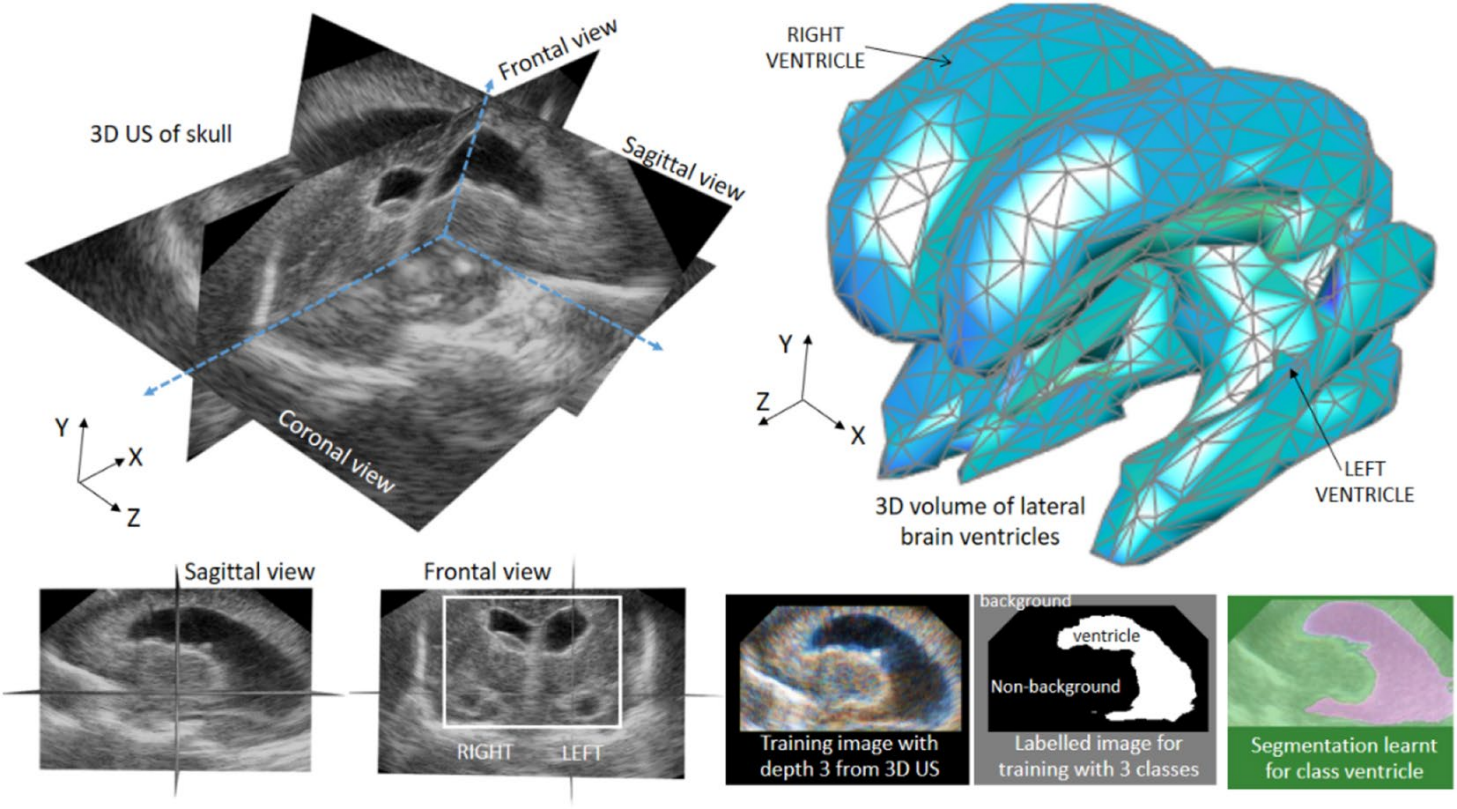
Left, orthoslice visualization of 3D US of the head of one infant with PHVD. The dilated ventricles are clearly visible. Top right, example of 3D patch surface visualization of the ventricles obtained using deep learning. Bottom left, representative sagittal and coronal slices of one 3D US of one patient with PHVD. Bottom right, representative RGB image generated by stacking three consecutive sagittal slices, the corresponding labels, and the segmentation calculated using a convolutional neural network (CNN).

Ideally, when performing supervised training, all classes would have an equal number of observations. However, the classes of the 3D US examined in this work are imbalanced, i.e. the class of pixels associated to ventricles is a minority class. Images have many more non-ventricle pixels (brain, cranium, background) than ventricle pixels because the former cover most of the area in the images. If not handled correctly, this imbalance is detrimental to the learning process because the learning is biased in favour of the dominant classes. For reducing the effects of this imbalance, we carried out three actions. First, instead of using every sagittal image of each 3D US, we used only those sagittal images contained within narrow rectangular regions of interest delineated manually on a coronal view prior segmentation (see white rectangle drawn on the coronal view of the 3D US in Fig. 8). In this way, imbalance between pixels with and without ventricle information is reduced. Second, we rebalanced the classes of the labels of the selected images by creating a new class “background” for those pixels that are exactly black. The resulting image labels contained three classes, thereby reducing the number of pixels of the non-ventricle classes while keeping intact the number of pixels of the ventricle class. Third, we used a reweighting scheme of each class in the cross-entropy loss as introduced in reference^16^; according to it, each pixel is weighted by α_c_ = m_p_/p_c_ where p_c_ is the number of pixels of class c divided by the total number of pixels in images where c is present, and m_p_ is the median of these frequencies. It has been shown empirically that using the computed weights in the loss function during training results in a sharp increase in class average accuracy and a slight increase in mean intersection over union, whereas the global accuracy decreases.

#### Fine-tuning the Segnet architecture on patients id1–id4

**Figure 9:**
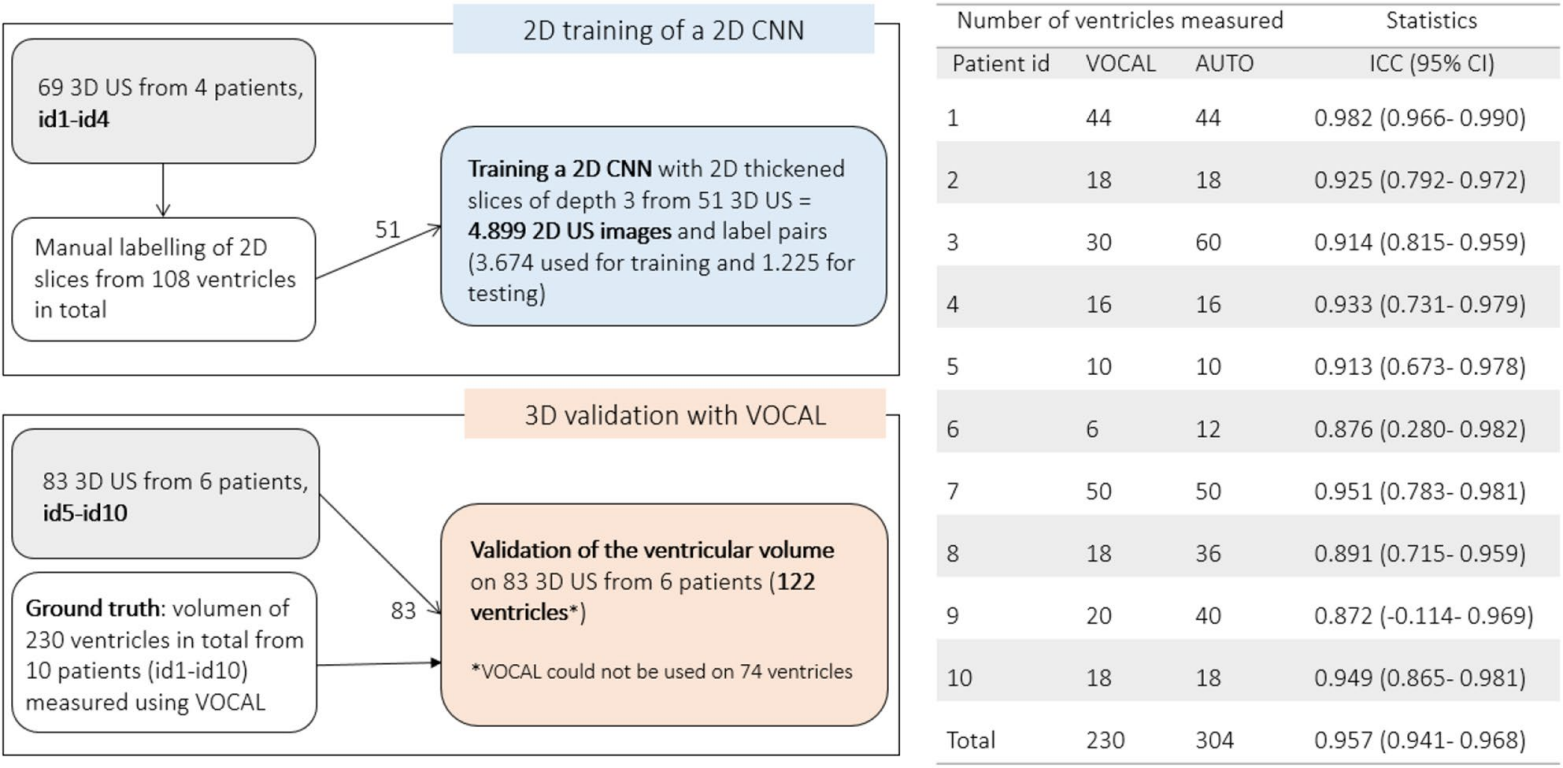
Left, description of the dataset and how it was distributed for training and testing the deep learning framework and for its validation against the gold standard (VOCAL). Right, the table shows the number of ventricles measured using VOCAL and/or the deep learning-based segmentation method and the intraclass coefficient (ICC) between both methods. Using VOCAL, there are situations in which the ventricles cannot be measured. For instance, in patients id3, id6, id8 and id9 the number of unmeasured ventricles reached 50% and the ICC is lower. Using deep learning, all ventricles from the ten patients could be measured.

For training the Segnet Network we did fine-tuning, thus the weights are initialized with weights from a model pretrained on ImageNet, and the final pixel classification layer is replaced with a new one. The network was trained using images from a selection of 3D US of four patients identified as id1, id2, id3, id4. Temporal series of 3D US were acquired from each patient resulting in 69 3D US. The graph in Fig. 9 summarizes how the data was used in this work. The training and the testing sets are the datasets of thickened 2D slices and ground truth labels used during the procedure of training the 2D CNN. The validation set refers to all the 3D US from which the ventricular volumes were measured quantitatively with both the automated segmentation procedure using the trained CNN and with VOCAL.

Overfitting is another important issue to consider when training a CNN. It can be avoided by using a large number of images during training. However, because many images in our dataset look similar we took additional measures to avoid overfitting as listed below.

1. We added image augmentation that included random x and y translation (± 20 pixels), rotation (± 30°), scale [0.8, 1.2], and x and y shear [± 20°].
2. L2 regularization of 0.0005 for the weights to the loss function was included.
3. We decreased the batch size to 2. The gradients calculated with a small mini batch are much noisier than gradients calculated with large batch size, so it is easier for the model to escape from sharp minimizers, and thus leads to better generalization^17^.
4. The CNN was trained using the stochastic gradient descent (SGD) with an initial learning rate of 0.01 and a momentum of 0.9. Training was performed with 4899 RGB images and 4899 labelled images that were divided into a training set and a test set using a 75:25 ratio. Training was carried out for ten epochs taking 9.3 h on a PC with a i3 CPU, 12 Mb of RAM and a GPU Nvidia Geforce 920MX. Before each epoch, the training set was shuffled and each mini batch (two images) was then chosen to ensure that each image is used only once in each epoch. The performance of the CNN for segmentation of the ventricle class was evaluated by measuring two common metrics: the average intersection over union (IoU) or Jaccard index that quantifies the percent overlap between the target mask and the prediction output of the CNN for all the 2D images of the test set.
5. The temporal series of 3D US were often taken one day apart and on some days it was difficult to see changes in the brain structure. We built the training dataset by selecting 52 3D US out of the 69 3D US of patients id1–id4 aiming to obtain a more representative training set of 3D US. For this, we followed the criteria of maximizing ventricular changes and leaving out of the training set those consecutive 3D US that did not show appreciable ventricular variations.

### Evaluation

#### Gold standard

We assessed our algorithm against the temporal series of volume measurements obtained using the Virtual Organ Computer-Aided Analysis (VOCAL) method (General Electric Medical Systems, Kretztechnik, Zipf, Austria) that is considered the gold standard. Using the same steps previously reported by our group^18–21^, we used a rotation angle of 15° with a starting plane of the view of the three horns of the lateral ventricle^18,20^, obtained once orthogonal planes were optimized and rotated to a position where the anteroposterior ventricular axis was perpendicular to the vertical rotation axis. Measurements were carried out through manual delimitation of the lateral ventricle contour in the 12 given planes. Once accomplished, the software presented the twelve planes of measurement for eventual contour corrections, automatically calculated the final volume, and rendered the respective ventricular surface. This method has been evaluated with high reliability and validity and it is considered the gold standard 3D US method for performing volumetric measurements^20–23^. Moreover, excellent intra and interobserver variability and reproducibility of this method of ventricular volume estimation had been previously investigated and published^18,20^.

#### Automatic 3D segmentation of ventricles

The volumes of the ventricles were measured as follows: at inference time, consecutive sagittal images were extracted iteratively from the 3D US and they were preprocessed following the same procedures used to build the training/test sets: (1) images with depth three were built by concatenating three consecutive sagittal slices; (2) the resulting RGB images were resized to 200 × 200 pixels using zero padding; and (3), the images were median filtered with a kernel [1, 3, 3]. Then, every image was passed through the trained CNN the output of which was the segmented slice. All the segmented sagittal slices of one 3D US were stacked to build a binary 3D volume setting to 1 only those pixels with labels of the ventricle class. At this stage, the Dice similarity coefficient (DSC) was used to quantify the percent overlap in 3D between the volumes of the ventricles segmented automatically and volumes of the ventricles segmented manually. The 3D visualization of the segmented ventricles was done using a patched surface with a limited number of 2000 patches. Finally, the volume of each ventricle was obtained by summing the voxels of each blob of the 3D volume and multiplying the sum by the calibrated voxel volume. These values are presented in the Figs. 2 and 3. In term of computational load, the average time needed for segmentation, analysis and visualization of one 3D ultrasound was 1.5 min.

### Statistical analysis

We performed a descriptive analysis and results were expressed by central tendency and dispersion measures for quantitative variables (mean, standard deviation, median and range); qualitative variables were expressed by frequency and percentage.

A bivariate analysis was performed to look for possible relationships between the variables. The Chi-square test was used for qualitative variables, and in case the requirements for this were not met, the exact Fisher test was used.

For the comparison of two means in the case of quantitative variables that follow a normal distribution, the Student t test was used and, in the case that they did not follow a normal distribution, a non-parametric test (Mann Withney–Wilcoxon’s U) was used.

We analysed the agreement of both methods (automatic and VOCAL) of ventricular volume trajectories through the average measure ICC for absolute agreement using two-way random effects model^24^. We calculated the DSC for measuring the degree of overlap in 3D between the ventricles segmented manually and automatically. We also performed Passing and Bablok regression as it allows for estimation of constant and proportional bias between two methods^25^. As the Passing–Bablok procedure should only be used on variables that are highly correlated we estimated Lin’s Concordance Correlation coefficient (CCC) of Absolute Agreement^26^. Moreover, we performed Bland–Altman (BA) analysis which allows a graphical representation of reproducibility. Thus, the agreement will be perfect if a line parallel to the abscissa axis is obtained and the ordinate is equal to zero. If the normality assumption is not rejected, 95% of the differences are expected to be included within the 95% agreement limit range. The quantitative analysis corroborates the bias observed in the graph and is better if the mean of the differences is closer to 0.

Statistical analysis was performed with the Stata 16.0 statistical package. A level of 95% (p < 0.05) was considered significant in all cases. All methods were carried out in accordance with relevant guidelines and regulations.
